# Is There a Future for Traditional Immunogens When We Have mRNA?

**DOI:** 10.3390/microorganisms11041004

**Published:** 2023-04-12

**Authors:** Karen K. Kyuregyan, Juris Jansons, Maria Isaguliants

**Affiliations:** 1 Central Research Institute of Epidemiology, 111123 Moscow, Russia; karen-kyuregyan@yandex.ru; 2 Scientific and Educational Resource Center for High-Performance Methods of Genomic Analysis, Peoples’ Friendship University of Russia (RUDN University), 117198 Moscow, Russia; 3 Latvian Biomedical Research and Study Centre, LV-1067 Riga, Latvia; jansons@biomed.lu.lv; 4 Department of Research, Riga Stradins University, LV-1007 Riga, Latvia

As the SARS-CoV-2 pandemic ends and we enter into a post-pandemic world, it is the time to reflect on the lessons learned. How do we respond to the next pandemic and what knowledge can we to transfer from this pandemic to the next one? One thing that appears obvious is that we will turn to an mRNA vaccine if the next infection outbreak is a previously unknown infectious agent that is threatening to become a pandemic. By now, with the SARS-CoV-2 experience and massive global introduction to mRNA vaccines, it may seem odd to even think of using proteins or other traditional immunogens. Vaccine developers worldwide are competing to come up with ingenious designs and clever delivery systems for mRNA, leaving behind other vaccine vehicles. Indeed, an mRNA vaccine is fast and easy to design in response to an emerging infection and can be produced using a generic and cost-reducing platform technology which, in theory, can be applied for all (or most) mRNA-based vaccines. However, one has to keep in mind few considerations.

First and foremost, mRNA vaccines must show equally good short- and long-term efficacy as protein-based vaccines. It may be argued that different platforms may have their own merits in driving different types of immune responses. The use of efficient and safe adjuvants may tip the balance in favor of protein-based vaccines and the possibility to tailor the desired type of immune response for a specific pathogen. Presently, little is known about the long-term efficacy of mRNA vaccines in humans in general, as the entire body of knowledge emanates from the experience with mRNA vaccines against SARS-CoV-2. Indeed, mRNA vaccines appear to have excellent short-term, but lower long-term efficacy and have to be continuously boosted. Whether this is an effect of the mRNA technology itself, or a property of the perpetually evolving viral target, remains to be determined. Their efficacy against the infectious agents with a profile different from the SARS-CoV-2 are also still unknown.

Secondly, while production may be in favor of mRNA-based vaccines, its logistics may become an obstacle since its stability is lower than that of traditional vaccines and requires cold-chain distribution. While the global roll out of mRNA vaccines during the COVID pandemic has proved that it is possible and has provided a network that new vaccines can benefit from, it still impacts the cost of vaccine administration. Novel mRNA formulations may improve vaccine stability and ultimately avoid the need for intact −80 °C cold chains, but these methodologies have yet to come.

Finally, the tremendous success of mRNA vaccines during the SARS-CoV-2 pandemic has paved the way for regulatory approval of mRNA vaccines and the way clinical trials are conducted. Still, mRNA vaccines are young and still unexplored with regard to their long-term safety and efficacy—there is much more accumulated safety knowledge on the use of protein-based vaccines. The statistics of massive mRNA application has yet to prove that they are as safe as traditional vaccines. 

All these aspects will have to be taken into account when moving forward with vaccine research and development. As shown by the work published in this Special Issue, despite mRNA vaccines’ success, R&D continues to actively explore other novel, as well as traditional, vaccine approaches. A significant share of the papers in this Special Issue is devoted to another novel vaccine modality, DNA vaccines, both in therapeutic and prophylactic applications, as well as to their delivery systems. Isaguliants et al. present a paper on the development of a therapeutic DNA vaccine against drug-resistant HIV-1 and showed that immunization of mice with DNA encoding resistant HIV-1 integrase induced potent T-cell responses and provided partial protection against integrase-expressing tumor cell growth in challenge experiments [1]. Jansons et al. present a study on the development of a therapeutic two-component DNA vaccine against HCV-related liver cancer, with one component encoding a viral nucleocapsid protein to target HCV-infected cells, and the other encoding telomerase reverse transcriptase to target tumor cells. Janson et al. demonstrated that simultaneous delivery of plasmids encoding these components abrogated the immune response against both proteins, indicating that there are drawbacks to simple combinations of components of multi-target DNA vaccines [2]. The third DNA vaccine study of this issue relates to the antibody response to an HIV-1 DNA vaccine after a booster with one or two protein components in vaccinees in a phase IIa HIV vaccine clinical trial. The results of this study proved the immunogenicity of DNA vaccines followed by protein boost. It also demonstrated that the second co-administered boosting component, HIV-1 subtype C envelope protein, provides no additional benefit in terms of frequency, breadth, or magnitude of antibody response which allows the simplification of vaccination protocols [3]. 

The focus of the studies by Tran et al. [4], Tuchynskaya et al. [5] and Kichatova et al. [6] was anti-viral antibody responses. Tran et al. developed a virus-like particle (VLP)-based vaccine on the backbone of the attenuated Kunjin strain of the West Nile virus and utilized it as a delivery system for the Ebola virus glycoprotein and the matrix protein genes. This multivalent system induced seroconversion for Ebola and West Nile virus proteins in mice, indicating the utility of such an approach both as a protein vaccine against West Nile virus and as a gene delivery system in the development of future Ebola vaccines capable of inducing strong protective antibody responses [4]. Using a mouse model, Tuchynskaya et al. analyzed which factors could potentially affect the efficacy of the inactivated whole-virion tick-borne encephalitis vaccine. This study showed that the decrease in neutralizing antibodies following the boost may be due to immunosuppression. The level of virus-neutralizing antibodies after the challenge was influenced by the ratio of non-infectious to infectious viral particles in the challenge virus; however, the latter had no effect on the protectivity [5]. Kichatova et al. surveyed the frequency and duration of the infection-induced antibody response to SARS-CoV-2 and assessed the diagnostic specificity of antibody tests and their application for monitoring of antibody responses to infection and to COVID-19 vaccines [6]. Among the findings of this study was the observation of a high degree of seropositivity to SARS-CoV-2 in non-vaccinated health care workers due to workplace exposure to the virus [6].

Delivery systems are a crucial part of vaccine development. Complementing the study by Tran et al. on the VLP-based Ebola vaccine [4], Petrovskis et al. [7] described another VLP-based delivery system, based on particles formed by the nucleocapsid protein of HBV (HBcAg). VLPs composed of HBcAg of different virus genotypes expressed in *E. coli* were used as potential carriers of mRNA vaccines as well as immunomodulating dsRNA [7]. To complement studies on DNA- [1,2,3], VLP- [4,7] and virion-based vaccines [5], Vasilyev et al. developed a classical vaccination approach based in the use of the attenuated virus [8]. This study investigated the capacity of a live attenuated influenza vaccine using viruses expressing truncated NS1 protein to protect mice against a challenge with a heterologous influenza virus. Vasilyev et al. showed that intranasal immunization with the NS1-truncated virus was associated with a stronger response against the T-cellular epitopes of the influenza virus and conferred better protection for the mice after challenge with a lethal viral dose compared to immunization with the wild-type H1N1 virus, which was attributed to a reduced inflammatory immune response in the lungs [8].

The breadth of vaccine research demonstrated in this Special Issue demonstrates that vaccinology not only dwell with new modalities, but also continues to develop classical approaches. Such diversification of vaccine resources is essential as one approach is not likely to be able to defend against every infectious disease that mankind will encounter in the future.

## Data Availability

Not applicable.
